# Temporal Drivers of Liking Based on Functional Data Analysis and Non-Additive Models for Multi-Attribute Time-Intensity Data of Fruit Chews

**DOI:** 10.3390/foods7060084

**Published:** 2018-06-03

**Authors:** Carla Kuesten, Jian Bi

**Affiliations:** 1 Amway, Ada, MI 49355, USA; 2 Sensometrics Research and Service, Richmond, VA 23236, USA; bbdjcy@aol.com

**Keywords:** relative importance of attributes to liking, multicollinearity, temporal drivers of liking (TDOL), functional data analysis, Choquet integral, fuzzy measure, multi-criteria decision-making, Shapley value, interaction indices, LMG statistic, multi-attribute time-intensity (MATI) data, fruit chews

## Abstract

Conventional drivers of liking analysis was extended with a time dimension into temporal drivers of liking (TDOL) based on functional data analysis methodology and non-additive models for multiple-attribute time-intensity (MATI) data. The non-additive models, which consider both direct effects and interaction effects of attributes to consumer overall liking, include Choquet integral and fuzzy measure in the multi-criteria decision-making, and linear regression based on variance decomposition. Dynamics of TDOL, i.e., the derivatives of the relative importance functional curves were also explored. Well-established R packages ‘fda’, ‘kappalab’ and ‘relaimpo’ were used in the paper for developing TDOL. Applied use of these methods shows that the relative importance of MATI curves offers insights for understanding the temporal aspects of consumer liking for fruit chews.

## 1. Introduction

Drivers of consumer liking is an important topic of sensory and consumer research. Drivers of liking can be defined as the attributes, which have the most important effects on overall liking [1]. Identification of the drivers is to determine relative importance of the explanatory attributes to liking in a model for sensory attributes and liking. However, determining relative importance of attributes to liking is a tricky task when two or more explanatory variables are nearly linearly dependent, i.e., multicollinearity, which are common situations for sensory attributes. There are some advanced mathematical and statistical techniques to measure relative importance of correlated attributes to liking considering both direct effects and interaction effects [2].

Relative importance of attributes to liking may also vary with time. Time-intensity is a dynamic sensory analysis methodology. It measures the intensity of a single sensory perception over time in response to a single exposure to a product or other sensory stimulus. The validity of the time-intensity measurement is based on the fact that sensory perception is a dynamic process and it should be measured dynamically [3]. An observation in conventional sensory analysis methods, such as quantitative descriptive analysis, is a single-point static rating—an integration by the respondent over the course of the sensory experience or in most practicing panels the highest intensity of the attribute at any given point in the experience, while it is a curve in a time-intensity measurement. Hence time-intensity measurement usually provides much more information about sensory properties of products than the conventional sensory analysis methods.

There are some proposed methods for simultaneous evaluations of multiple sensory attributes in time-intensity evaluation, such as the dual-attribute time-intensity method [4], the Temporal Dominance of Sensations method [5], and the multiple-attribute time-intensity (MATI) method [6]. MATI data in a fruit chews experiment are used in this study.

Temporal drivers of liking (TDOL) is a hot topic in sensory and consumer fields in recent years. See, e.g., [7,8,9,10] in recent sensory literature for the topic. The current TDOL methods in the literature mentioned above focus on Temporal Dominance of Sensations data.

### 1.1. Non-Additive Model for Modeling Panel Descriptive Attributes and Consumer Liking

Multiple regression and other conventional regressions are often used to model consumer liking and sensory attributes. A common assumption for the models (additive models) is that the regression variables are independent of each other. Under this assumption, the models are additive models. This assumption, however, does not hold in many situations. Sensory attributes are usually dependent. It is noted that even if the assumption is not true, the model can be used for prediction of liking without serious difficulty. However, it seriously affects the determination of relative importance of sensory attributes to liking, i.e., determination of drivers of liking [2]. The non-additive models, which consider both direct effects and interaction effects of attributes to consumer liking, are needed to model the correlated sensory attributes and liking.

In the past about two decades, a non-additive model—Choquet integral and fuzzy measure—has been developed and used in the multi-criteria decision-making (also called multi-criteria decision aiding) field and other fields for modeling data in situations where interaction occurs for multiple criteria (attributes). This model is a special regression model. The main advantage compared to the conventional regression models is that this model is able to take into account interactions and dependence between sensory attributes. It can not only provide the predictions of liking, but also give Shapley values, for relative importance of sensory attributes, and interaction measures of each pair of the sensory attributes. See, e.g., [11] for more details about the methodology. Shapley values were proposed by Shapley [12] in game theory. Alfonso [13] introduced the methodology in the sensory field. The R package ‘kappalab’, which stands for ‘laboratory for capacities’, can be used for the methodology. See, e.g., [14] for the R package.

### 1.2. Non-Additive Model for Modeling Both Consumer Attributes and Consumer Liking

There are some different non-additive models for determination of relative importance of correlated attributes. One is the LMG statistic [2,15] and R package ‘relaimpo’ [16,17]. The LMG statistic is based on linear regression but with variance decomposition, i.e., averaging over orderings of attributes in the model. The LMG statistic was first proposed by Lindeman, Merenda, and Gold [18] and henceforth is named. We can use this model for determination of the relative importance of attributes for both consumer liking and consumer attributes including just-about-right attributes.

### 1.3. Relative Importance Curves and Temporal Drivers of Liking

Functional data analysis techniques and the R package ‘fda’ and code ‘wfda’ can be used on the multiple sets of relative importance measures (Shapley, interaction and LMG statistic) for a series of time points based on the non-additive models to produce the functional curves of relative importance measures.

### 1.4. Dynamic Aspects of Temporal Drivers of Liking

The dynamic aspects of temporal drivers of liking are the derivatives of the relative importance functional curves, which reflect rates of change of the relative importance measure. Functional data analysis offers direct access to derivatives of functional curves by using a built-in function ‘deriv.fd’ in the R package ‘fda’.

The main objective of this study is to provide a novel TDOL technique which is based on some advanced statistical technologies and is used for MATI data. The TDOL involves, respectively, panel attributes with continuous scales; panel attributes with CATA (check-all-that-apply) scales; and consumer attributes with continuous and just-about-right scales. Functional data analysis [19,20,21] and non-additive models, i.e., Choquet integral and fuzzy measure in the multi-criteria decision-making [11,13,14], and LMG statistic based on variance decomposition in linear regression [15], are used for the TDOL for MATI data. Well-established R packages ‘fda’, ‘kappalab’ and ‘relaimpo’ for the advanced mathematical and statistical methodologies were used for analysis of the MATI data and TDOL in the paper. The R packages can be downloaded freely from http://cran.r-project.org. Some R codes (‘kapf’, ‘relaf20’, ‘wfda’) used in the paper can be found in the Supplementary File S1in the online version of this paper.

## 2. Materials and Methods

This research guidance study involves development work on fruit chews using both trained sensory descriptive panel and consumer MATI data collected throughout consumption. The functional ingredients of this fruit chew that provide health benefits pose taste and texture challenges. The fruit components (acerola cherry, baobab, hesperidin, and other citrus bioflavonoids) deliver unpleasant flavors—bitter, astringency, and tannic qualities. The fruit fiber from the acerola cherry creates a fibrous, gritty texture with particulates that require a lot of unpleasant mouth clean-up. Given that the flavor and texture experience during chew-down is particularly relevant for consumer acceptance of this functional food form, MATI was used to help us characterize the sensory flavor and texture profile of the chew.

The objectives of this research were focused on product development and analytical methods. Product understanding and guidance was sought toward optimizing consumer acceptance. Descriptive and consumer results were gathered to understand consumer liking as a function of sensory descriptive and consumer attributes throughout consumption. The analytical research goals were to expand multivariate statistical analyses for MATI data and to extend the conventional drivers of liking analysis with a time dimension to TDOL.

### 2.1. Descriptive Panel

Nine descriptive panelists were selected that had completed over 100 h of descriptive training using universal scale attribute standards. Universal flavor aromatic reference standards included soda cracker, Mott’s applesauce, Minute Maid orange juice, and Welch’s grape juice, with intensity ratings of 2, 5, 7 and 10, respectively. The panelists had demonstrated proficiency in terms of lexicon understanding and usage, repeatability, ability to discriminate, scale usage, agreement, and reproducibility prior to participation. Panelists were practiced and proficient in MATI evaluations prior to the study. A pace of 3 s between attributes was used during MATI data collection.

### 2.2. Consumer Panel

Forty adults who consume chews regularly were selected from among company employees. These consumers ranged in age from 22 to 55 and represented an equal number of males and females. Respondents were screened based on frequency of consumption of chews (product category users), acceptability to the flavors tested, willingness to taste test the chews, no dental issues, non-smokers, normal odor sensitivity (no colds or problems with smelling during the test period), and no known food allergies. All were given a 1-h one-on-one orientation to the MATI program and practiced with a warm-up run. The facilitator of the session made sure that each respondent understood the purpose of the study, demonstrated good eye–hand coordination for data collection, and correctly entered their results. The sample ratings collected from the consumers included: hedonic flavor and texture ratings as well as intensity ratings. Each sample varied in the total time it took respondents to reach complete dissolution (~90 s) and was followed by a 1-min aftertaste. A 2-min break between samples was imposed. Use of in-house panels is routine and well-practiced in industry and considered relevant at early stages of new product development. While a larger sample size would in general be more appropriate for testing with consumers, this smaller sample size of *N* = 40 is justified given that the prototypes being tested are preliminary efforts and exploratory in nature. Product development was looking for direction and guidance at this stage.

### 2.3. Test Samples

Three test samples were presented; all cherry fruit chews. A retail competitor (Sample C) was used as a warm-up sample. In addition to the functional active ingredients mentioned above, the two test prototypes contained date and raisin paste, pineapple fruit juice concentrate, gum Arabic, fruit solids, sugar cane fiber, magnesium phosphate, humectants, emulsifiers, acids and natural flavors. The prototypes evaluated differed in amount of acerola cherry fiber, described as Sample A (textured prototype) and Sample B (smooth prototype). The cherry fiber content contributed to differences in Smoothness. All samples were square and of similar shape and size (~5.4 gms per piece, 2 pieces per serving). Test samples were produced in the lab, stored at room temperature, and evaluated at one month of age (~30 days old). The retail competitor was purchased in a local grocery and within shelf-life.

### 2.4. Study Design

#### 2.4.1. Descriptive Panel

**Figure 1:**
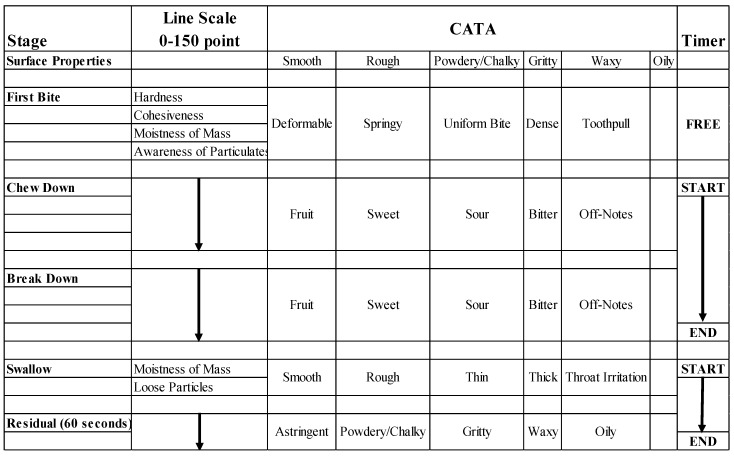
Multiple-attribute time-intensity (MATI) descriptive panel scorecard. CATA, Check-All-That-Apply.

The panel evaluated the chews through 6 stages of the ‘Texture Breakdown Path’ [22] as illustrated in Figure 1, using 2 chews/test sample. The blind-coded prototype samples were randomized and balanced across sessions. The first chew was used to evaluate surface properties and the first bite. A second chew was used through chew-down and the remaining stages. This allowed recording onset and duration for each stage and events such as ‘stuck on teeth’ and the moment when the chew was completely dissolved. During evaluation of the first chew for surface properties and first bite, panelists were on ‘free time’ (time used was captured but panelists were allowed to record ratings at their own pace). Initiating chew-down with the second chew re-started the timer thru to swallow, pacing panelists at 3-s per attribute. Swallowing triggered restart of the timer again and began the 60-s aftertaste. The CATA items were checked if presented at an intensity of 3 or higher on a 0–15 point structured line scale, not as dominate sensation as in Temporal Dominance of Sensations. The line scale data was captured in tenths of a unit for a total of 0–150 points.

#### 2.4.2. Consumer Panel

Consumers evaluated flavor and texture in 2 separate sessions; in addition, 2 separate sessions for each modality were used to apply different scales. For flavor, the following attributes were captured: Overall Liking of Flavor, Fruit Flavor, Sweetness, Sourness and Pleasantness of Aftertaste; for texture, the following attributes: Overall Liking of Texture, Chewiness Stickiness, Smoothness and Pleasantness of Mouthfeel. Ratings were collected as hedonic and intensity line scales or (in separate sessions) as hedonic and just-about-right category scales. Each consumer completed a total of 4 test sessions, each session approximately 20 min in duration—2 flavor and 2 texture sessions completing 3 samples, 1 rep per session. A cross-over design was applied to balance order of modality as well as scale type across the consumers; the prototype samples were randomized and balanced across serving positions.

### 2.5. MATI Data Analysis

Statistical analyses developed and applied for this work are extensive. Functional data analysis was used to smooth and present the data graphically. Functional data analysis supports analysis of curves and is particularly applicable to analysis of TI data. Using functional data analysis, we predicted the values of the sensory attributes or liking. Two different approaches (depending on the scale type) were applied. For line scales, relative importance (Shapley values) were estimated using Choquet integrals and fuzzy measures in multi-criteria decision-making. This is a special regression model that can take into account interaction and dependence between sensory attributes. For category scales, 9-point hedonic and just-about-right scales, relative importance values (LMG statistics) were estimated using linear regression based on variance decomposition which takes into account correlated attributes and uses averaging over orders [18]. R software was used for the analyses. Further details and examples follow that illustrate the techniques applied to MATI data.

## 3. Results

**Figure 2:**
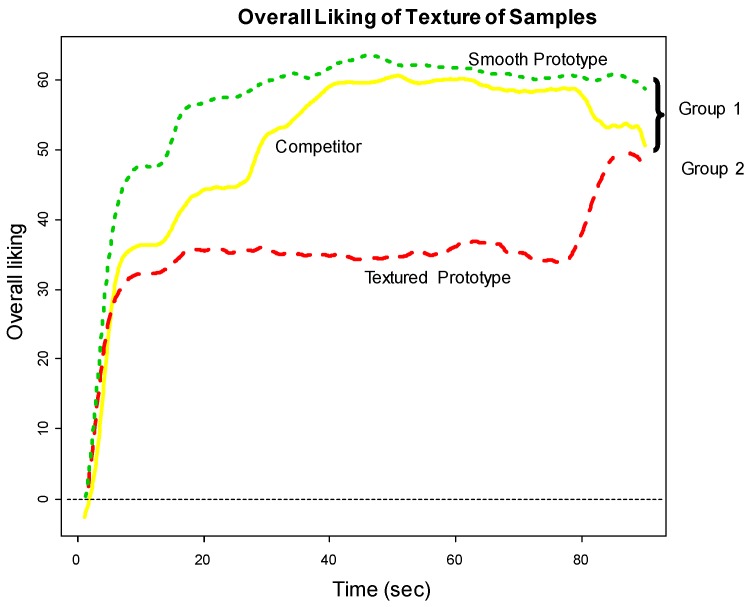
Consumer chew-down Overall Liking of Texture.

**Figure 3:**
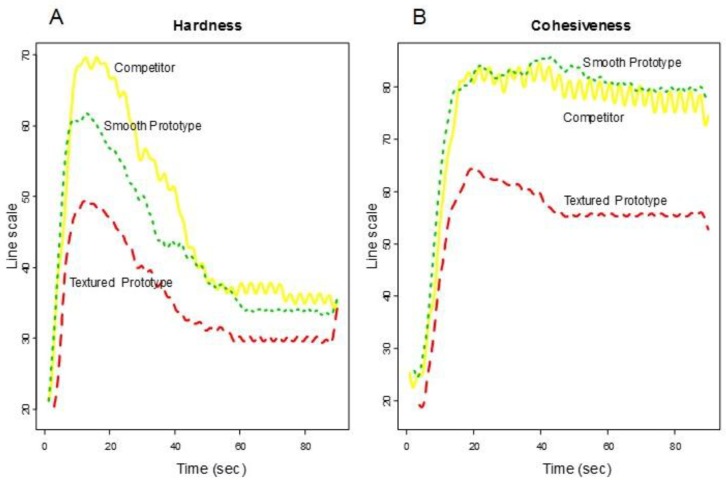
Descriptive panel chew-down (**A**) Hardness and (**B**) Cohesiveness.

**Figure 4:**
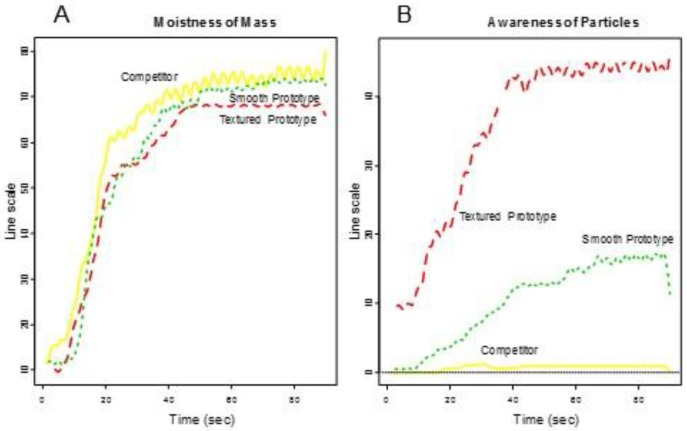
Descriptive panel chew-down (**A**) Moistness of Mass and (**B**) Awareness of Particles.

**Figure 5:**
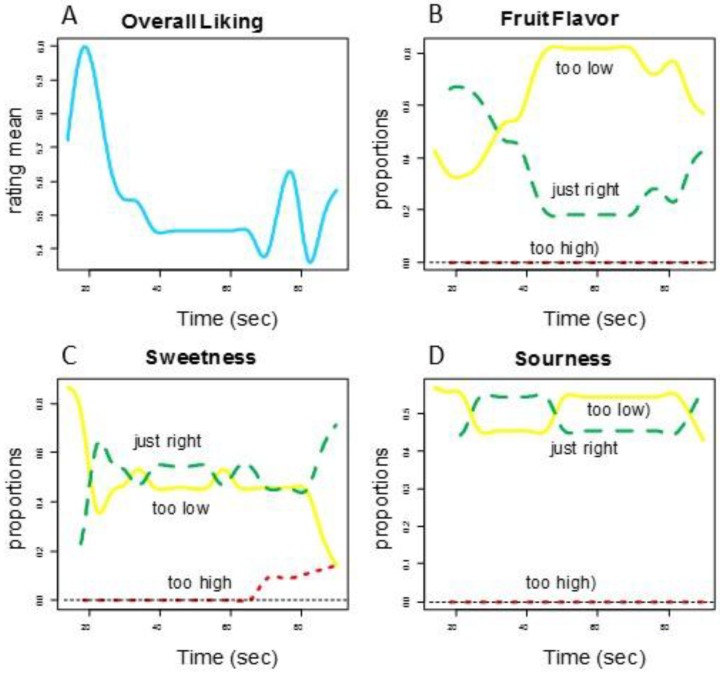
Consumer panel chew-down—smooth prototype (**A**) Overall Liking of Flavor, (**B**) Fruit Flavor, (**C**) Sweetness, and (**D**) Sourness just-about-right scales.

Consumers’ rated Overall Liking of Texture higher for the smooth prototype and the competitor (Group 1) and lower for the textured prototype (Group 2) (See Figure 2). Figure 3 shows the functional curves for the 2 sensory attributes Hardness and Cohesiveness for the 3 samples. Initially, the competitor is rated hardest, the textured prototype softest and the smooth prototype in-between all peaking around 10-s. The decay curve is relatively steep for all samples, but slightly more gradual for the textured prototype. The samples finish similarly. The competitor and smooth prototype rate similarly and highest over time on Cohesiveness. Though we see more variation with the competitor, the textured prototype is recognized as less cohesive. Figure 4 shows both Moistness of Mass and Awareness of Particles for the 3 samples, again showing more similarity between the competitor and the smooth prototype versus the less moist textured prototype. In addition, we see the obvious issue of particulates for the textured prototype during chew-down. In terms of flavor, Figure 5 shows that consumers indicate their liking decreases over time; they want more Fruit Flavor, Sourness and Sweetness (especially initially and at the end). The proportions of “too strong” are almost zero at any time. The proportions of ‘just right’ and ‘too low’ seem to mirror each other—and cross-over/switch around 35 s for intensity of Fruit Flavor, 20 and 80 s for Sweetness and back and forth during 20–80 s for Sourness. Further work is required to optimize the flavor of the smooth prototype.

The results below cover even more depth of understanding for measuring, analyzing and interpreting the presence, duration, change (derivative) and rate of change (second derivative) of attributes in relation to temporal drivers of liking. Understanding how best to leverage these insights and bring them to use within product development optimization will require further study and development efforts.

### 3.1. Non-Additive Model for Modeling Panel Descriptive Attributes and Consumer Liking

The Shapley value, a measure of relative importance, is calculated for each sensory attribute in the R package ‘kappalab’. A matrix is given for interaction effects for each pair of the attributes. The sum of the Shapley values for the attributes is one. A positive interaction value in the interaction matrix suggests that the pair of criteria are complementary, while a negative value suggests that they are substitutive.

#### 3.1.1. Numerical Results Output 1 (Panel Attributes with Line Scale)

For the MATI descriptive panel data of the fruit chews experiment, the relative importance values of the 4 panel sensory intensity attributes with line scales (Hardness, Cohesiveness, Moistness of Mass, and Awareness of Particles) to consumer Overall Liking of Texture, and interaction of a pair of the sensory attributes were obtained for each given time point in the range of 0 and 90 s using the R package ‘kappalab’. For example, for the 50th second time point, the results are given in Supplementary File S2 using the R code ‘kapf’.

In this example, the largest Shapley value is 0.41 for Hardness. The interaction between Hardness and MoistnessOfMass is positive (0.21) and hence the two attributes are complementary, while interaction between Cohesiveness and Awareness of Particles is negative (−0.17), and hence the two attributes are substitutive.

For some time points, e.g., for the 30 time points from 3 to 90 s in a step of 3 s, we estimated the 30 sets (each set is a matrix of 3 × 4) of the values of the 4 panel sensory attributes for 3 samples from the corresponding 3 sets of the functional curves. We also estimated the 30 sets (each set is a vector with a length of 3) of Overall Liking of Texture from the 3 curves of Overall Liking of Texture. With the estimated values for each time point, we calculated the Shapley values of the 4 sensory attributes, which are the relative importance values of the 4 attributes to Overall Liking of Texture at that time point. With the data for 30 time points, we obtained 30 sets of the Shapley values interaction indices for the 4 attributes, which are listed in Table 1 and Table 2.

#### 3.1.2. Numerical Results Output 2 (Panel Attributes with CATA Scale)

In the panel MATI experiment, there is a ‘check-all-that-apply’ (CATA) question with 5 CATA items (UniformBite, Deformable, Dense, Springy, and Toothpull) in First Bite. We determined the relative importance of each CATA item to consumer Overall Liking of Texture at a specified time point. The data for each item is a proportion. The R package ‘kappalb’ and code ‘kapf’ were used. See Supplementary File S3.

For example, for time at 5 s, the relative importance values, i.e., the Shapley values for the 5 CATA items, are obtained below. It suggests that the item UniformBite is the most important among the 5 items to Overall Liking of Texture at 5 s with Shapley value of 0.42. The interaction effect between Deformable and UniformBite is −0.47. The negative interaction suggests that the two attributes are substitutive. 

For each time point from 1 to 25 s, we obtained the data, i.e., the proportions of each of the 5 CATA items for the 3 products, and rating means of consumer Overall Liking of Texture at that time. Hence, we derived the 25 sets of Shapley values for the CATA items, which are listed in Table 3.

### 3.2. Non-Additive Model for Modeling Both Consumer Attributes and Consumer Liking

For each time point, we generated a data matrix with n rows (consumer panelists) and k + 1 columns for Overall Liking and k attributes. We then calculated relative importance of the attributes in terms of the LMG statistic, which is a measure of relative importance as a Shapley value. The LMG statistic is equivalent to the Shapley value in game theory [12]. See e.g., [15] for the relationship between LMG and the Shapley value.

#### Numerical Results Output 3 (Consumer Just-About-Right Attributes with 3 Levels)

For the consumer MATI data in the fruit chews experiment, we determined the relative importance of 3 consumer just-about-right attributes (Fruit Flavor, Sweetness and Sourness) to Overall Liking of Flavor for Sample B. The just-about-right attributes are treated as factors with 3 levels (−1 = too weak, 0 = just about right, +1 = too strong). For example, for the time point at 14 s, the values of relative importance for the 3 just-about-right attributes Fruit Flavor, Sweetness, and Sourness are 0.27, 0.64 and 0.08, respectively. At time 60 s, the relative importance values are 0.64, 0.12 and 0.24, respectively. At time 90 s, the values are 0.54, 0.33 and 0.24, respectively. Obviously, the relative importance of the attributes to Overall Liking of Flavor varies with time. See Supplementary File S4 for analysis and results.

We calculated the LMG values of the 3 just-about-right attributes at 20 time points (14 to 90 s with a step of 4) for Sample B, which are listed in Table 4. It is noted that using linear regression with variance decomposition, i.e., averaging over orderings, it provides only relative importance values (e.g., LMG) of the attributes to liking, it does not provide the interaction indices as that in the fuzzy measure and Choquet integral in numerical output 1.

### 3.3. Relative Importance Curves and Temporal Drivers of Liking

For multiple sets of relative importance measures (Shapley, interaction and LMG) that we have obtained in Table 1, Table 2, Table 3 and Table 4 for a series of time points based on non-additive models, we produced the functional curves of relative importance measures by using functional data analysis techniques and the R package ‘fda’ and code ‘wfda’. The functional objects for the relative importance measures based on the observed data in Table 1, Table 2, Table 3 and Table 4 were produced. See Supplementary File S5.

#### 3.3.1. Numerical Results Output 1 

For the functional object ‘rivaluefd’, we generated the relative importance functional curves. See code in Supplementary File S6 used to generate the plot.

**Figure 6:**
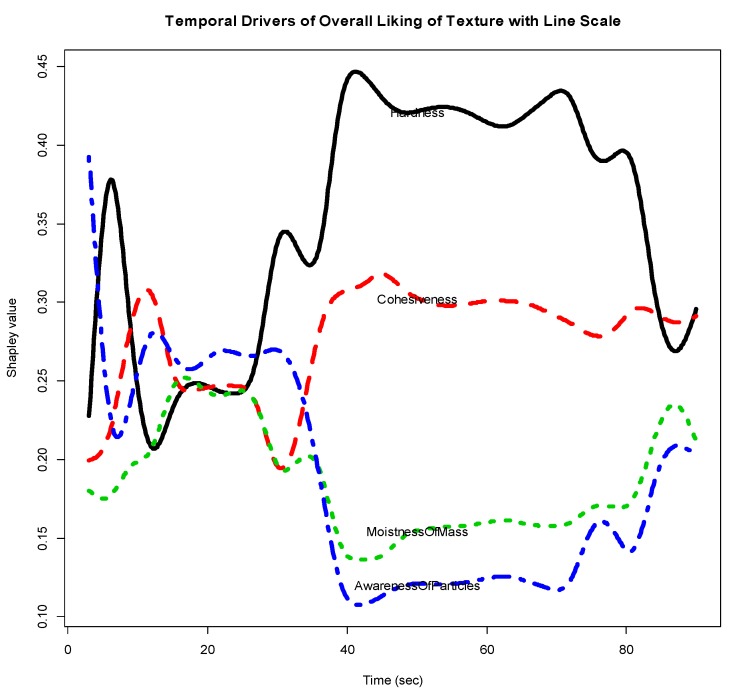
Temporal drivers of Overall Liking of Texture with line scale.

Figure 6 gives the Shapley curves of the 4 sensory attributes, which suggest the TDOL. From Figure 6, we see that after about 30 s, the attribute Hardness is the driver of Overall Liking of Texture. In the range from 1 to 30 s, the situation is mixed and unstable.

**Figure 7:**
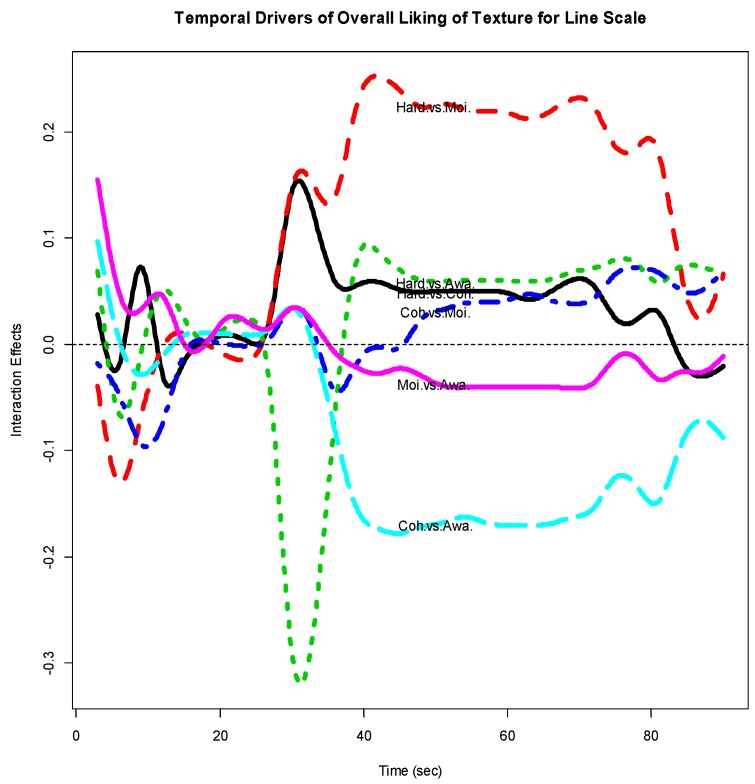
Temporal drivers of Overall Liking of Texture with line scale (interaction).

Figure 7 gives the interaction effect curves for each of the 6 pairs of the 4 sensory attributes based on the functional object ‘intvaluefd’. It seems that after about 40 s, the interaction effects between *Hardness* and each of the other attributes, and between Cohesiveness and MoistnessOfMass are consistently positive, i.e., complementary, while Cohesiveness vs. AwarenessOfParticles and MoistnessOfMass vs. AwarenessOfParticles are consistently negative, i.e., substitutive.

#### 3.3.2. Numerical Results Output 2 

**Figure 8:**
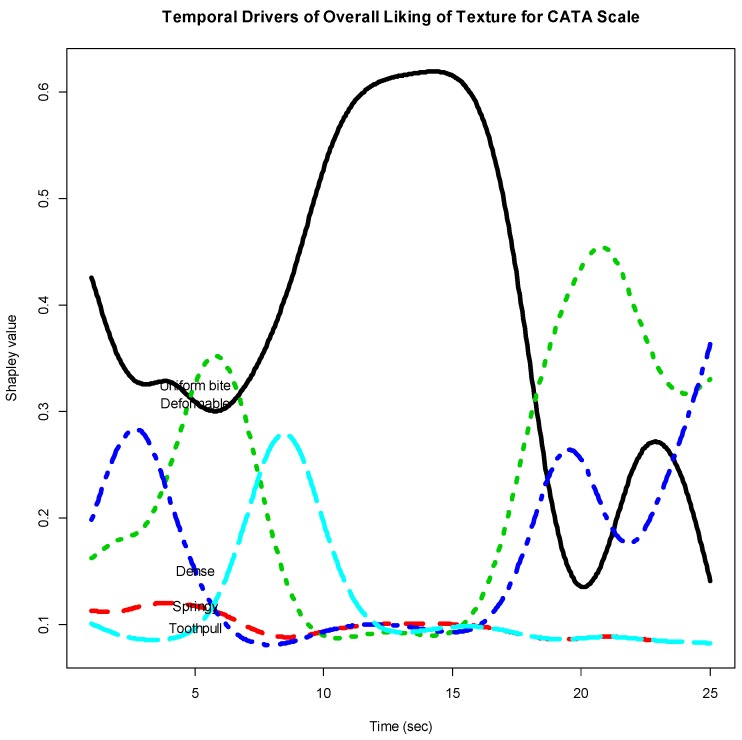
Temporal drivers of Overall Liking of Texture with CATA scale (5 CATA items in the first bite).

Figure 8 gives the Shapley curves of the 5 CATA items based on the functional object ‘rivalue2fd’. The curves reflect temporal relative importance of the CATA items to Overall Liking with time. It should be mentioned that the interaction indices could also be obtained.

#### 3.3.3. Numerical Results Output 3 

**Figure 9:**
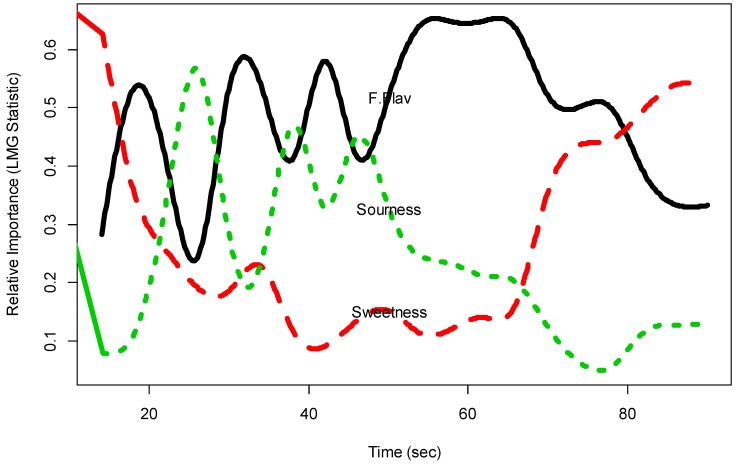
Temporal drivers of Overall Liking of Texture with just-about-right scale.

Figure 9 gives the LMG curves of the 3 just-about-right attributes to Overall Liking of Flavor based on the functional object ‘jarRI.fd’. The curves in Figure 9 show possible temporal drivers of liking analysis for just-about-right attributes for both consumer liking and consumer just-about-right attributes. It suggests that the relative importance of the 3 just-about-right attributes to Overall Liking of Flavor varies with time. In the beginning and the end (after about 80 s), Sweetness seems the most important effect on liking within the 3 attributes, while during most of the time (about 30 to 80 s), Fruit Flavor is the driver of Overall Liking.

### 3.4. Dynamic Aspects of Temporal Drivers of Liking

The first and second derivatives of the functional objects in Figure 6, Figure 7, Figure 8 and Figure 9 reflect rates of change of the relative importance measures. These are obtained as shown in Supplementary File S7.

**Figure 10:**
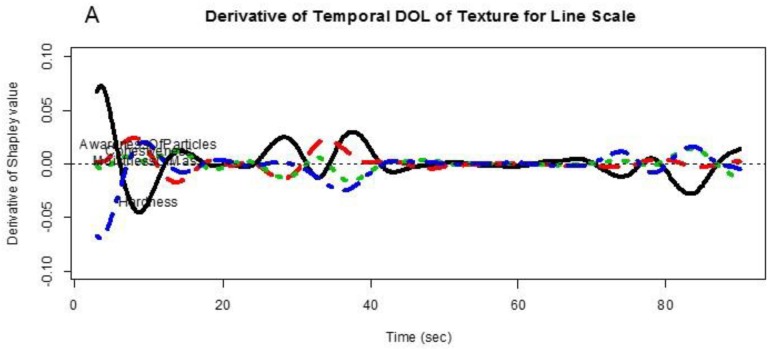

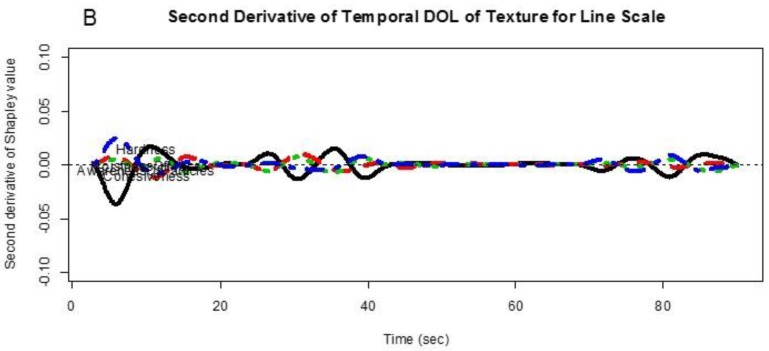
Derivatives of temporal drivers of Overall Liking of Texture with line scale. (**A**) First Derivative and (**B**) Second Derivative.

We produced the charts of the first and second derivatives of the function object, e.g., ‘rivaluefd’; it is shown in Figure 10. See Supplementary File S7.

Figure 10 roughly shows the first and second derivatives of the temporal DOL curves in Figure 6 for the numerical outputs 1–2 in this paper.

The key of this exploration is about the practical meaning and interpretation for derivatives of the relative importance functional curves. The first and second derivatives of the TDOL curves show the velocities and accelerations of the relative importance measures. The values of zero in the first derivative of the relative importance measure correspond to local maximum or minimum values of the relative importance measures. If the absolute values of the first and second derivatives for an attribute are close to zero, it suggests that the relative importance of the attribute to liking is stable.

## 4. Discussion

This research was pursued due to a concern about consumer texture acceptability; the fruit chews under investigation provided an undesirable lack of cohesiveness and moistness with high awareness of particulates during chew-down due to the fiber content. Research efforts were undertaken to determine if modifications could be made to improve the texture experience for consumers. Evidence of the improvement is highlighted with the use of MATI TDOL, wherein we see that the initial prototype (textured prototype) demonstrates less Cohesiveness and Moistness during chew-down compared to a modified prototype (smooth prototype) which more closely approximates the competitor for Overall Liking of Texture.

### 4.1. MATI Product Research

Practical, applied learnings for product development and optimization have been gleaned from this work. This study has shown that MATI offers the ability to relate sensory and consumer data on a time-dependent basis. Conventional drivers of liking DOL analyses can be extended with a time dimension into TDOL. MATI is a natural extension of traditional time-intensity with one attribute. This method serves as a rapid ‘real-time’ assessment tool for product screening and optimization. As shown here, it was applied successfully for gathering information throughout the Texture Breakdown Path wherein consumption events were logged. Training and practice are required for reliable results; an easy user-interface is important. MATI can serve to aid and enhance panel training experiences. In regard to consumer research with MATI, care should be taken to explain the method thoroughly. How MATI programming is structured for data collection impacts the results. Results may vary from conventional methods.

### 4.2. MATI Data Analytics

Relative importance of attributes to consumer liking may vary with time. The curves of relative importance of attributes reflect the variations, and hence the conventional drivers of liking were extended into TDOL based on functional data analysis and non-additive models, which consider both direct and indirect effects of an attribute to consumer liking.

There are different non-additive models for determination of relative importance of attributes to liking. The Choquet integral and fuzzy measure in the multi-criteria decision-making are suitable to determination of panel descriptive attributes to *Overall Liking*, in which the number of samples are usually small. The linear regression based on variance decomposition is suitable to determination of both consumer attributes and consumer liking, i.e., original consumer data, because in this method, the number of consumer panelists (rows in the data file) should be larger, at least larger than the number of attributes (columns of the data file).

The dynamic aspects of relative importance curves of attributes may provide additional insights for TDOL. We found at which time points the relative importance of an attribute to liking tends to change, i.e., rates of change (first derivative) and acceleration of change (second derivative). Functional data analysis is a powerful tool for exploring the dynamic aspect of the TDOL. 

### 4.3. Future Research

Sensory and consumer temporal methods have grown in recent years. Future efforts to continue this evolution will undoubtedly involve ongoing comparison of simultaneous time-intensity results with conventional descriptive panel, discrete time-intensity, and consumer data, enhanced experiences for MATI data collection, investigation into variations on protocols, instructions, warm-up, conditioning or priming of initial responses, examination of the impact of interface and design on response ratings, as well as application and extension of MATI to other product categories. Furthering the understanding of consumer temporal multivariate data through analytics and how best to leverage the dynamic aspects of TDOL is on the horizon.

## 5. Conclusions

The objectives of this research were satisfied through use of MATI with descriptive and consumer data of fruit chews for product research guidance. The work also accomplished the research goals of successfully expanding multivariate statistical analyses for MATI data and extending the conventional drivers of liking analysis with a time dimension to TDOL.

## Figures and Tables

**Table 1:** Shapley values for each panel descriptive attribute to Overall Liking of Texture varying with time (‘rivalue’).

Second	Hardness	Cohesiveness	Moistness Of Mass	Awareness Of Particles
3	0.21	0.20	0.18	0.40
6	0.41	0.21	0.17	0.21
9	0.27	0.29	0.20	0.24
12	0.20	0.31	0.20	0.29
15	0.24	0.25	0.25	0.26
18	0.25	0.25	0.25	0.26
21	0.24	0.25	0.24	0.27
24	0.25	0.24	0.24	0.27
27	0.25	0.24	0.24	0.26
30	0.35	0.19	0.19	0.27
33	0.33	0.22	0.20	0.25
36	0.32	0.29	0.20	0.19
39	0.44	0.31	0.14	0.12
42	0.44	0.31	0.14	0.11
45	0.43	0.32	0.14	0.11
48	0.42	0.31	0.15	0.12
51	0.42	0.30	0.16	0.12
54	0.42	0.30	0.16	0.12
57	0.42	0.30	0.16	0.12
60	0.41	0.30	0.16	0.12
63	0.41	0.30	0.16	0.13
66	0.42	0.30	0.16	0.12
69	0.43	0.29	0.16	0.12
72	0.44	0.29	0.16	0.12
75	0.39	0.28	0.17	0.16
78	0.39	0.28	0.17	0.16
81	0.40	0.30	0.17	0.13
84	0.30	0.29	0.21	0.19
87	0.27	0.29	0.24	0.21
90	0.30	0.29	0.21	0.20

**Table 2:** Interaction indices for each pair of panel descriptive attributes to Overall Liking of Texture varying with time (‘intvalue’).

Second	“HvC”	“HvM”	“HvA”	“CvM”	“CvA”	“MvA”
3	0.04	−0.03	0.08	−0.02	0.10	0.16
6	−0.06	−0.15	−0.09	−0.04	0	0.04
9	0.13	−0.06	0	−0.1	−0.03	0.03
12	−0.07	0	0.06	−0.08	−0.01	0.06
15	0	0.01	0.02	0	0.01	−0.01
18	0	0	0	0	0.01	0
21	0.01	−0.01	0.02	0	0.01	0.03
24	0.01	−0.01	0.01	0	0.01	0.02
27	0	0.01	0	0	0.01	0.01
30	0.17	0.17	−0.33	0.04	0.04	0.04
33	0.12	0.15	−0.27	0.02	0	0.02
36	0.05	0.12	−0.06	−0.06	−0.08	−0.01
39	0.06	0.24	0.09	−0.01	−0.16	−0.02
42	0.06	0.25	0.08	0	−0.17	−0.03
45	0.05	0.24	0.07	−0.01	-0.18	−0.02
48	0.05	0.22	0.06	0.03	−0.17	−0.03
51	0.05	0.23	0.06	0.03	−0.17	−0.04
54	0.05	0.22	0.06	0.04	−0.16	−0.04
57	0.05	0.22	0.06	0.04	−0.17	−0.04
60	0.05	0.22	0.06	0.04	−0.17	−0.04
63	0.04	0.21	0.06	0.05	−0.17	−0.04
66	0.05	0.22	0.06	0.04	−0.17	−0.04
69	0.06	0.23	0.07	0.04	−0.16	−0.04
72	0.06	0.23	0.07	0.04	−0.16	−0.04
75	0.02	0.18	0.08	0.07	−0.12	−0.01
78	0.02	0.18	0.08	0.07	−0.13	−0.01
81	0.04	0.20	0.05	0.07	−0.16	−0.04
84	−0.02	0.06	0.08	0.05	−0.09	−0.02
87	−0.03	0.02	0.07	0.05	−0.07	−0.03
90	−0.02	0.07	0.07	0.07	−0.09	−0.01

“HvC”: Hardness versus Cohesiveness; “HvM”: Hardness versus MoistnessOfMass; “HvA”: Hardness versus AwarenessOfParticles; “CvM”: Cohesiveness versus MoistnessOfMass; “CvA”: Cohesiveness versus AwarenessOfParticles; “MvA”: MoistnessOfMass versus AwarenessOfParticles.

**Table 3:** Shapley values for each panel descriptive CATA item to Overall Liking of Texture varying with time (‘rivalue2’).

Second	Deformable	Springy	UniformBite	Dense	Toothpull
1	0.48	0.12	0.14	0.14	0.11
2	0.28	0.08	0.28	0.28	0.08
3	0.25	0.13	0.10	0.43	0.09
4	0.49	0.12	0.14	0.14	0.11
5	0.26	0.11	0.42	0.12	0.09
6	0.26	0.13	0.42	0.10	0.09
7	0.35	0.09	0.35	0.09	0.13
8	0.38	0.08	0.08	0.08	0.37
9	0.38	0.08	0.08	0.08	0.37
10	0.60	0.10	0.10	0.10	0.10
11	0.60	0.10	0.10	0.10	0.10
12	0.60	0.10	0.10	0.10	0.10
13	0.60	0.10	0.10	0.10	0.10
14	0.60	0.10	0.10	0.10	0.10
15	0.60	0.10	0.10	0.10	0.10
16	0.60	0.10	0.10	0.10	0.10
17	0.60	0.10	0.10	0.10	0.10
18	0.38	0.08	0.37	0.08	0.08
19	0.08	0.08	0.37	0.37	0.08
20	0.08	0.08	0.37	0.37	0.08
21	0.10	0.10	0.6	0.10	0.10
22	0.37	0.08	0.38	0.08	0.08
23	0.28	0.08	0.28	0.28	0.08
24	0.28	0.08	0.28	0.28	0.08
25	0.08	0.08	0.37	0.37	0.08

**Table 4:** LMG values for each consumer just-about-right attribute to Overall Liking of Flavor (Sample B) varying with time (‘jarLMG’).

Second	Fruit Flavor	Sweetness	Sourness
14	0.27	0.64	0.08
18	0.55	0.35	0.11
22	0.43	0.26	0.31
26	0.20	0.19	0.61
30	0.56	0.18	0.26
34	0.55	0.24	0.21
38	0.37	0.11	0.52
42	0.63	0.09	0.28
46	0.38	0.13	0.48
50	0.53	0.16	0.31
54	0.65	0.11	0.25
58	0.64	0.12	0.24
62	0.65	0.15	0.21
66	0.65	0.15	0.21
70	0.51	0.37	0.12
74	0.50	0.44	0.06
78	0.51	0.44	0.05
82	0.38	0.50	0.12
86	0.33	0.54	0.13
90	0.33	0.54	0.13

## Supplementary Materials

The following are available online at http://www.mdpi.com/2304-8158/7/6/84/s1. Supplementary File S1: R Codes, Supplementary File S2: Numerical Results Output 1 (panel attributes with line scale), Supplementary File S3: Numerical Results Output 2 (panel attributes with CATA scale), Supplementary File S4: Numerical Results Output 3 (Consumer JAR Attributes with 3 Levels), Supplementary File S5: Relative importance curves and temporal drivers of liking, Supplementary File S6: Numerical Results Output 1 (Shapley values plot), Supplementary File S7: Dynamic aspects of temporal drivers of liking.
