# Supplementary material for: Temporal Drivers of Liking Based on Functional Data Analysis and Non-Additive Models for Multi-Attribute Time-Intensity Data of Fruit Chews
Source: Foods. 2018 Jun 3;7(6):84. doi: 10.3390/foods7060084 (PMC6025064; doi:10.3390/foods7060084)
Supplement: Supplementary file 1 [file foods-07-00084-s001.zip › Supplementary File S1.docx]

Temporal Drivers of Liking Based on Functional Data Analysis and Non-Additive Models for Multi-Attribute Time-Intensity Data of Fruit Chews

Carla Kuesten ^1,^* and Jian Bi ^2^

Supplementary File S1: R Codes

#1

kapf<-function(x){

#using R package "kappalab"

x<-as.matrix(x)

k<-dim(x)[2]

mu.unif<-as.capacity(uniform.capacity(k-1))

kap<-heuristic.ls.capa.ident(k-1,mu.unif,x[,2:k],x[,1])

#kap<-least.squares.capa.ident(k-1,2,x[,2:k],x[,1])

#cat("ok","\n")

nam<-dimnames(x)[[2]][2:k]

cat(nam,"\n")

sp<-Shapley.value(kap$solution)

spp<-cbind(seq(1,k-1),sp)

dimnames(spp)<-list(nam,c("No.","Shapley"))

spp<-spp[rev(sort.list(spp[,2])),]

int<-round(interaction.indices(kap$solution),2)

dimnames(int)<-list(nam,nam)

sp0<-cbind(seq(1,k-1),sp)

dimnames(sp0)[[1]]<-nam

dotchart(sp0[sort.list(sp0[,2]),2], main="Relative Importance",xlab="Shapley Value")

#vv<-kapv(x[,2:k],x[,1])

write.table(round(spp,2),"c:\\temMD\\shapley",sep=",")

#list(Shapley=spp,Interaction=int,FuzzyMeasure=kap$solution)

list(Shapley=spp,Interaction=int)

}

#2

relaf20<-function(x){

#R package "relaimpo"

x<-as.data.frame(x)

k<-dim(x)[2]-1

for(i in 1:k){x[,1+i]<-as.factor(x[,1+i])}

nam<-dimnames(x)[[2]][2:(k+1)]

lmg<-lm(OvLik~.,data=x)

m<-calc.relimp(lmg,type=c("lmg"),rela=T)

plot(m,names.abbrev=8)

m

}

#3

wfda<-function (x)

{#for fda data

k1<-dim(x)[1]

p<-dim(x)[2]

bas<-create.bspline.basis(c(x[1,1],x[k1,1]),k1+2,4,x[,1])

fdpar<-fdPar(bas,2,lambda=10^0.01)

y.fd<-smooth.basis(x[,1],x[,2:p],fdpar)$fd

y.fd

}
