# Supplementary material for: Temporal Drivers of Liking Based on Functional Data Analysis and Non-Additive Models for Multi-Attribute Time-Intensity Data of Fruit Chews
Source: Foods. 2018 Jun 3;7(6):84. doi: 10.3390/foods7060084 (PMC6025064; doi:10.3390/foods7060084)
Supplement: Supplementary file 1 [file foods-07-00084-s001.zip › Supplementary File S2.docx]

Temporal Drivers of Liking Based on Functional Data Analysis and Non-Additive Models for Multi-Attribute Time-Intensity Data of Fruit Chews

Carla Kuesten ^1,^* and Jian Bi ^2^

Supplementary File S2: Numerical Results Output 1 (panel attributes with line scale)

>library(kappalab)

> kapf(ridatf(50))

hlms: L2 ending value of criterion: 6.29965 at iteration: 108

Hardness Cohesiveness MoistnessOfMass AwarenessOfParticles

$Shapley

No. Shapley

Hardness 1 0.4080246

Cohesiveness 2 0.3054231

MoistnessOfMass 3 0.1600822

AwarenessOfParticles 4 0.1264702

$Interaction

Hardness Cohesiveness MoistnessOfMass AwarenessOfParticles

Hardness NA 0.04 0.21 0.05

Cohesiveness 0.04 NA 0.04 -0.17

MoistnessOfMass 0.21 0.04 NA -0.03

AwarenessOfParticles 0.05 -0.17 -0.03 NA

> ridatf(50)

Ovlik Hardness Cohesiveness MoistnessOfMass AwarenessOfParticles

s 60.45786 38.14606 77.86109 71.01760 0.8063203

a 34.36890 31.18682 55.48813 68.04092 43.1561871

b 62.49207 38.07207 83.74212 70.72280 12.7547871
