# Supplementary material for: Temporal Drivers of Liking Based on Functional Data Analysis and Non-Additive Models for Multi-Attribute Time-Intensity Data of Fruit Chews
Source: Foods. 2018 Jun 3;7(6):84. doi: 10.3390/foods7060084 (PMC6025064; doi:10.3390/foods7060084)
Supplement: Supplementary file 1 [file foods-07-00084-s001.zip › Supplementary File S3.docx]

Temporal Drivers of Liking Based on Functional Data Analysis and Non-Additive Models for Multi-Attribute Time-Intensity Data of Fruit Chews

Carla Kuesten ^1,^* and Jian Bi ^2^

Supplementary File S3: Numerical Results Output 2 (panel attributes with CATA scale)

> kapf(ridatf2(5))

hlms: L2 ending value of criterion: 29.3379 at iteration: 499

Deformable Springy UniformBite Dense Toothpull

$Shapley

No. Shapley

UniformBite 3 0.41503552

Deformable 1 0.26490233

Dense 4 0.12336885

Springy 2 0.10668738

Toothpull 5 0.09000592

$Interaction

Deformable Springy UniformBite Dense Toothpull

Deformable NA -0.04 -0.47 0.03 -0.11

Springy -0.04 NA -0.18 -0.05 0.02

UniformBite -0.47 -0.18 NA -0.21 -0.14

Dense 0.03 -0.05 -0.21 NA -0.02

Toothpull -0.11 0.02 -0.14 -0.02 NA

> ridatf2(5)

Ovlik Deformable Springy UniformBite Dense Toothpull

s 24.87133 0.0331310740 -1.438299e-06 -7.125958e-04 -7.872343e-04 3.258800e-07

a 26.31004 -0.0001228212 0.000000e+00 8.724403e-05 2.395766e-05 -1.718994e-06

b 35.67287 0.0051528890 1.545209e-05 -8.829827e-04 2.520816e-05 -2.502394e-06
