# Supplementary material for: Temporal Drivers of Liking Based on Functional Data Analysis and Non-Additive Models for Multi-Attribute Time-Intensity Data of Fruit Chews
Source: Foods. 2018 Jun 3;7(6):84. doi: 10.3390/foods7060084 (PMC6025064; doi:10.3390/foods7060084)
Supplement: Supplementary file 1 [file foods-07-00084-s001.zip › Supplementary File S4.docx]

Temporal Drivers of Liking Based on Functional Data Analysis and Non-Additive Models for Multi-Attribute Time-Intensity Data of Fruit Chews

Carla Kuesten ^1,^* and Jian Bi ^2^

Supplementary File S4: Numerical Results Output 3 (Consumer JAR Attributes with 3 Levels)

>library(relaimpo)

> jardatf(14)[,2:5]

OvLik Fruit.Flav Sweetness Sourness

2 5 0 -1 -1

3 8 0 0 0

5 5 -1 -1 0

6 5 -1 -1 -1

7 5 -1 -1 0

8 7 0 -1 -1

10 5 0 -1 -1

> relaf20(jardatf(14)[,2:5])

Response variable: OvLik

Total response variance: 1.571429

Analysis based on 7 observations

3 Regressors:

Fruit.Flav Sweetness Sourness

Proportion of variance explained by model: 71.72%

Metrics are normalized to sum to 100% (rela=TRUE).

Relative importance metrics:

lmg

Fruit.Flav 0.27364678

Sweetness 0.64372756

Sourness 0.08262566

> jardatf(60)[,2:5]

OvLik Fruit.Flav Sweetness Sourness

1 9 0 0 0

2 4 -1 0 -1

3 6 -1 -1 0

4 2 -1 0 0

5 6 -1 -1 0

6 5 -1 -1 -1

7 4 -1 -1 -1

8 9 -1 0 0

9 2 -1 -1 -1

10 8 0 0 -1

11 5 -1 -1 -1

> relaf20(jardatf(60)[,2:5])

Response variable: OvLik

Total response variance: 6.072727

Analysis based on 11 observations

3 Regressors:

Fruit.Flav Sweetness Sourness

Proportion of variance explained by model: 49.14%

Metrics are normalized to sum to 100% (rela=TRUE).

Relative importance metrics:

lmg

Fruit.Flav 0.6445744

Sweetness 0.1202889

Sourness 0.2351367

> jardatf(90)[,2:5]

OvLik Fruit.Flav Sweetness Sourness

1 9 0 0 0

2 4 -1 0 -1

3 6 0 0 0

4 2 -1 0 0

5 9 -1 1 0

6 5 0 0 -1

7 4 -1 -1 -1

> relaf20(jardatf(90)[,2:5])

Response variable: OvLik

Total response variance: 6.952381

Analysis based on 7 observations

4 Regressors:

Some regressors combined in groups:

Group Sweetness : Sweetness0 Sweetness1

Relative importance of 3 (groups of) regressors assessed:

Sweetness Fruit.Flav Sourness

Proportion of variance explained by model: 75.34%

Metrics are normalized to sum to 100% (rela=TRUE).

Relative importance metrics:

lmg

Sweetness 0.5372348

Fruit.Flav 0.3328030

Sourness 0.1299621
