# Supplementary material for: Temporal Drivers of Liking Based on Functional Data Analysis and Non-Additive Models for Multi-Attribute Time-Intensity Data of Fruit Chews
Source: Foods. 2018 Jun 3;7(6):84. doi: 10.3390/foods7060084 (PMC6025064; doi:10.3390/foods7060084)
Supplement: Supplementary file 1 [file foods-07-00084-s001.zip › Supplementary File S6.docx]

Temporal Drivers of Liking Based on Functional Data Analysis and Non-Additive Models for Multi-Attribute Time-Intensity Data of Fruit Chews

Carla Kuesten ^1,^* and Jian Bi ^2^

Supplementary File S6: Numerical Results Output 1 (Shapley values plot)

plot(rivaluefd, xlab="Time (sec)",ylab="Shapley value", main="Temporal Drivers of Overall Liking of Texture with Line Scale",lwd=5)

text(50,eval.fd(50,rivaluefd)[1],"Hardness")

text(50,eval.fd(50, rivaluefd)[2],"Cohesiveness")

text(50,eval.fd(50, rivaluefd)[3],"MoistnessOfMass")

text(50,eval.fd(50, rivaluefd)[4],"AwarenessOfParticles")
