# Supplementary material for: Temporal Drivers of Liking Based on Functional Data Analysis and Non-Additive Models for Multi-Attribute Time-Intensity Data of Fruit Chews
Source: Foods. 2018 Jun 3;7(6):84. doi: 10.3390/foods7060084 (PMC6025064; doi:10.3390/foods7060084)
Supplement: Supplementary file 1 [file foods-07-00084-s001.zip › Supplementary File S7.docx]

Temporal Drivers of Liking Based on Functional Data Analysis and Non-Additive Models for Multi-Attribute Time-Intensity Data of Fruit Chews

Carla Kuesten ^1,^* and Jian Bi ^2^

Supplementary File S7: Dynamic aspects of temporal drivers of liking

deriv.fd(rivaluefd)

deriv.fd(rivaluefd,2)

deriv.fd(intvaluefd)

deriv.fd(intvaluefd,2)

deriv.fd(rivalue2fd)

deriv.fd(rivalue2fd,2)

deriv.fd(jarRI.fd)

deriv.fd(jarRI.fd,2)

par(mfrow=c(2,1))

plot(deriv.fd(rivaluefd), xlab="Time (sec)",ylab="Derivative of Shapley value", main=" Derivative of Temporal DOL of Texture for Line Scale",lwd=5,ylim=c(-0.1,0.1))

text(10,eval.fd(10,deriv.fd(rivaluefd))[1],"Hardness")

text(10,eval.fd(10, deriv.fd(rivaluefd))[2],"Cohesiveness")

text(10,eval.fd(10, deriv.fd(rivaluefd))[3],"MoistnessOfMass")

text(10,eval.fd(10, deriv.fd(rivaluefd))[4],"AwarenessOfParticles")

plot(deriv.fd(rivaluefd,2), xlab="Time (sec)",ylab="Second derivative of Shapley value", main="Second Derivative of Temporal DOL of Texture for Line Scale",lwd=5,ylim=c(-0.1,0.1))

text(10,eval.fd(10,deriv.fd(rivaluefd,2))[1],"Hardness")

text(10,eval.fd(10, deriv.fd(rivaluefd,2))[2],"Cohesiveness")

text(10,eval.fd(10, deriv.fd(rivaluefd,2))[3],"MoistnessOfMass")

text(10,eval.fd(10, deriv.fd(rivaluefd,2))[4],"AwarenessOfParticles")
